# Circulating Micro-RNAs Predict the Risk of Recurrence in Triple-Negative Breast Cancer

**DOI:** 10.3390/cells13221884

**Published:** 2024-11-14

**Authors:** Jouni Kujala, Maria Tengström, Sami Heikkinen, Mari Taipale, Veli-Matti Kosma, Jaana M. Hartikainen, Arto Mannermaa

**Affiliations:** 1 Institute of Clinical Medicine, Clinical Pathology and Forensic Medicine, University of Eastern Finland, FI-70211 Kuopio, Finland; jouni.kujala@uef.fi (J.K.); veli-matti.kosma@uef.fi (V.-M.K.); 2 Cancer Center, Department of Oncology, Kuopio University Hospital, FI-70029 Kuopio, Finland; maria.tengstrom@pshyvinvointialue.fi; 3 Institute of Biomedicine, School of Medicine, University of Eastern Finland, FI-70211 Kuopio, Finland; sami.heikkinen@uef.fi (S.H.); mari.taipale@uef.fi (M.T.); 4 A. I. Virtanen Institute for Molecular Sciences, University of Eastern Finland, FI-70211 Kuopio, Finland; 5 Biobank of Eastern Finland, Kuopio University Hospital, FI-70029 Kuopio, Finland; 6 Genome Center of Eastern Finland, Institute of Clinical Medicine, University of Eastern Finland, FI-70211 Kuopio, Finland; 7 Multidisciplinary Cancer Research Community (Cancer RC), University of Eastern Finland, FI-70211 Kuopio, Finland

**Keywords:** liquid biopsy, sequencing, serum, survival

## Abstract

Triple-negative breast cancer (TNBC) is an aggressive subtype of breast cancer with a high tendency for developing a recurrent disease. Circulating micro-RNAs (cmiRNAs) obtained through liquid biopsy are potential prognostic biomarkers for the assessment of TNBC recurrence risk. In this study, we sequenced cmiRNAs from the serum samples of 14 recurrent and 19 non-recurrent TNBC cases and compared expression profiles in relation to recurrence status, survival data and miRNA expression in matched tumor samples. Differential expression analysis between recurrent and non-recurrent cases identified ten differentially expressed (DE) cmiRNAs, of which cmiRNAs miR-21-5p (*p* = 0.030, HR = 1.87, 95% CI 1.06–3.30), miR-16-5p (*p* = 0.032, HR = 0.53, 95% CI 0.30–0.95), and miR-26b-5p (*p* = 0.023, HR = 0.52, 95% CI 0.29–0.91) were associated with recurrence-free survival in multivariable survival analysis. Expression profiles of matched tumor and serum samples were shown to correlate with each other. DE cmiRNAs were associated with common cancer-related signaling pathways and improved the overall predictive performance of the logistic regression model assessing the recurrence risk. Our results indicate that recurrent and non-recurrent TNBC differ in their cmiRNA expression profiles, and that three specific cmiRNAs can be used to assess the risk of recurrence in TNBC.

## 1. Introduction

Triple-negative breast cancer (TNBC) is a heterogenous subtype of breast cancer (BC) that is characterized by the lack of estrogen receptor, progesterone receptor expression, along with normal expression levels of human epidermal growth factor receptor 2 (HER2). TNBC accounts for approximately 10–20% of all invasive BC cases [1,2,3] and has been associated with poor prognosis until the recent introduction of immunotherapies (ITs) in neoadjuvant and adjuvant setting [4]. However, not all patients benefit from ITs, and these treatments are also associated with the risk of developing irreversible endocrine disorders [5,6].

Although ITs are shown to decrease the recurrence rate of TNBC, the subtype is known for its high tendency to develop distant metastases [5,6,7]. Different prediction models have been introduced to estimate the risk of recurrent disease and distant metastases, but these models tend to have poor predictive performance for TNBC when compared to other BC subtypes [8,9,10]. The histopathological examination of tumor biopsies has remained the gold standard both for the diagnosis of TNBC and estimating its invasiveness. However, traditional tumor biopsies may not be representative of the entire tumor due to tumor heterogeneity, and procedures to collect biopsies are invasive and not always feasible in the clinical setting [11]. Moreover, there are no comprehensively validated biomarkers that would accurately predict the TNBC metastasis, and novel predictive biomarkers for metastatic TNBC are therefore needed to identify high-risk TNBC cases earlier.

Micro-RNAs (miRNAs) are noncoding RNAs that are involved in the post-transcriptional regulation of genes by inhibition of translation or mRNA cleavage [12]. The dysregulated expression of miRNAs and their direct impact on cancer-associated signaling pathways have been documented in a wide range of cancer diseases, and miRNAs are generally considered potential cancer biomarkers [13]. Recent studies have demonstrated that cancer cells release miRNAs to the extracellular environment through active secretion and cell death, thus suggesting the potential use of circulating miRNAs (cmiRNAs) as non-invasive biomarkers that could be used to predict the outcomes of BC [14,15,16,17].

Here, we isolated and sequenced circulating miRNAs from the serum samples of 33 TNBC cases to identify circulating miRNAs that are potentially associated with recurrent TNBC and could be potentially used as prognostic biomarkers to predict the clinical outcome of TNBC patients.

## 2. Materials and Methods

### 2.1. Patient Cohorts and Sample Material

This study included a cohort of 33 Eastern Finnish BC patients who had been diagnosed with a TNBC without locoregional or distant metastases at the time of diagnosis (Table 1). The cohort was collected to include both patients who remained recurrence-free (19 patients) and those who developed locoregional recurrence or distant metastases (14 patients) during the first five years of follow-up. Clinical data and sample materials were obtained from the Kuopio Breast Cancer Project (KBCP), which is a prospective population-based case-control study conducted in 1990–1995 in Eastern Finland [18,19,20]. To assess the impact of long-term freezing on cmiRNA expression levels, three serum samples from the more recent Itä-Länsi rintasyöpäprojekti (ILRS), a prospective population-based breast cancer study conducted in 2010 to 2014, were used as a control to investigate the effect of storage time.

Analyzed serum samples were collected (1) at the time of diagnosis prior to the initiation of treatment, (2) after the end of curative treatment, and (3) at the latest follow-up examination prior to the diagnosis of recurrence or distant disease. Serum samples with similar sampling intervals were selected for cases who remained recurrence-free. All serum samples were stored in −70 °C. Matched fresh-frozen tumor samples from the primary tumors were available for 25 TNBC cases. All tumor samples were obtained during cancer surgery before the initiation of curative treatment. All tumor samples were immediately covered with optimum cutting temperature compound after resection, cooled in liquid isopentane and liquid nitrogen, and stored in −70 °C.

### 2.2. Isolation of cmiRNAs and Tumor RNA

Circulating miRNAs were isolated from patient serum samples with a miRNEasy Serum Plasma Kit (Qiagen, Hilden, Germany) according to the manufacturer’s protocol. The total RNA from FF tumor samples was isolated with an Ambion mirVana miRNA Isolation Kit (Life Technologies, Carlsbad, CA, USA) according to the manufacturer’s protocol. The purity and concentration of isolated RNA samples were assessed with a NanoDrop ND-1000 spectrophotometer (Thermo Fisher Scientific, Waltham, MA, USA).

### 2.3. Library Preparation and Sequencing

All cmiRNA libraries were prepared with a QIAseq miRNA Library Kit (Qiagen) according to the manufacturer’s protocol. The concentration and size of the cmiRNA libraries was confirmed with the Agilent 2100 Bioanalyzer (Agilent Technologies, Santa Clara, CA, USA) with the High-Sensitivity DNA Kit (Agilent Technologies) and Qubit 2.0 fluorometer (Thermo Fisher Scientific) with the DNA High-Sensitivity assay (Invitrogen, Walthman, MA, USA). Libraries were sequenced on the Illumina NextSeq platform (Illumina, San Diego, CA, USA) with 75 bp reads.

Tumor miRNA libraries were prepared with a TruSeq Small RNA Library Preparation Kit (Illumina) according to the manufacturer’s protocol as previously described [21]. The concentration, size and quality of the tumor miRNA libraries was confirmed, and the sequencing was performed as previously described [21]. Total RNA-seq libraries were constructed from 800 ng of total RNA. First, ribosomal RNA was removed using the NEBNext rRNA Depletion Kit (New England BioLabs, Ipswich, MA, USA). Then, libraries were prepared as instructed using the NEBNext Ultra II Directional RNA Library Prep Kit for Illumina (New England BioLabs). The amount of library produced was measured with the Qubit 2.0 fluorometer using a DNA High-Sensitivity assay, and its quality was checked with the Agilent Bioanalyzer using the DNA 1000 kit (Agilent Technologies). After indexing, the libraries were combined and sequenced on the Illumina NextSeq 500 platform (Illumina) with 75 bp reads.

### 2.4. Bioinformatics

Sequencing data from cmiRNA-seq and miRNA-seq were processed with the in-house bioinformatics pipeline built with the Snakemake workflow management system (v.7.18.2) [22]. Unique molecular identifier (UMI) sequences were extracted and added to FASTQ header with UMI tools (v.1.1.4) [23] while discarding the 3′ adapter and primer sequences. Poor-quality reads and reads shorter than 16 bp were discarded with cutadapt (v.4.2) [23]. Trimmed reads were first aligned to human rRNA and tRNA sequences obtained from RFAM database (v.14) [24]. Unaligned reads were then aligned to human mature miRNA sequences obtained from the miRbase (v.22) [23] with Bowtie2 (v.2.5.1) [25]. Aligned reads were converted to BAM format with Samtools (v.1.16.1) [23] and deduplicated with UMI tools. Raw read counts were counted with the Samtools idxstats command. The bioinformatic analysis of serum and tumor samples slightly differed in terms of trimming and deduplication steps, as the sequenced tumor RNA libraries did not utilize UMI sequences.

The 75-nucleotide single-end RNA-seq reads were quality controlled using FastQC (v.0.11.7) [26]. Reads were then trimmed with Trimmomatic (v.0.39) [27] to remove Illumina sequencing adapters and poor-quality read ends, using the following as essential settings: ILLUMINACLIP:2:30:10, SLIDINGWINDOW:4:10, LEADING:3, TRAILING:3, MINLEN:50. Reads aligning to mtDNA or rRNA, the phiX174 genome, or composed of a single nucleotide were removed using STAR (v.2.7.9a) [28]. The remaining reads were aligned to the GENCODE human transcriptome version 38 (for genome version hg38) using STAR (v.2.7.9a) with essential non-default settings: --seedSearchStartLmax 12, --alignSJoverhangMin 15, --outFilterMultimapNmax 100, --outFilterMismatchNmax 33, --outFilterMatchNminOverLread 0, --outFilterScoreMinOverLread 0.3, and --outFilterType BySJout. The unstranded, uniquely mapping, gene-wise counts for primary alignments produced by STAR were collected in R (v.4.2.2) using Rsubread::featureCounts (v.2.12.3) [29].

Differential expression analyses were carried out using the DESeq2 R package (v.1.38.0) [30] with the Wald statistical test corrected for the multiple testing using the Benjamini–Hochberg correction. A false discovery rate (FDR) of ≤0.05 was considered as statistically significantly differentially expressed (DE). Variance stabilizing transformation (VST) and reads per million (RPM) transformations were used for statistical analyses and visualization, respectively.

### 2.5. Statistical Analyses

Survival data were analyzed with the univariable log–rank and Kaplan–Meier estimators followed by the multivariable Cox proportional hazard’s model. The used covariates in the multivariable survival analyses were age at the time of diagnosis, tumor grade, tumor stage, chemotherapy, and radiotherapy. Recurrence-free survival (RFS) was calculated as the time of diagnosis to the time of first local or distant metastasis or new BC. The overall survival (OS) and breast cancer-specific survival (BCSS) were calculated as the time from the date of diagnosis to the date of last follow-up or death where the cause of death was coded either caused by BC or not caused by BC. For all survival analyses, a *p*-value of ≤0.05 was considered as statistically significant. Survival analyses were conducted using IBM SPSS Statistics v.26 software (IBM).

To investigate the potential association between DE miRNAs and tumor features, all patients were divided into four groups based on the quartiles of miRNA expression. A Chi-squared test was used to test whether the distribution of categorical variables differs from the expected distribution. A *p*-value of ≤0.05 was considered as a statistically significant association. The prognostic value of DE cmiRNAs was further evaluated with univariable and multivariable logistic regression analyses. The variables’ age at the time of diagnosis, tumor grade and stage, and nodal status were used to build a reference multivariable regression model to predict the occurrence of recurrent disease. VST-normalized cmiRNA expressions were added to the reference model either as they were or as a composite variable obtained from the first principal component (PC1) of principal component analysis. The diagnostic performance of the models was estimated with a receiver operating characteristic (ROC) analysis and k-fold cross-validation. Bootstrapping with replacement was used to calculate the area under the curve (AUC) metric and its 95% confidence interval. A Chi-squared test and ROC analysis were conducted using the Scipy (v.1.11.0) [31] and scikit-learn (v.1.4.2) [32] Python packages.

### 2.6. Pathway Enrichment Analysis

The DIANA-mirPath (v.4) online tool with the Tarbase (v.8) database of experimentally validated miRNA–target gene interactions (MTIs) was used to identify known direct MTIs and affected biological pathways [33,34]. An FDR of ≤0.05 was considered as statistically significant pathway enrichment. The COSMIC Cancer Gene Census database (v.96) was used to identify MTIs that affect known oncogenes and tumor suppressors [35]. Identified MTIs were further analyzed and visualized with Cytoscape (v.3.10.1) software [36].

To validate the biological relevance of identified MTIs, we calculated Pearson correlation coefficient for the cmiRNA and tumor mRNA expression values of each MTI. A *p*-value of ≤0.10 was considered as statistically significant correlation. Hypergeometric gene set enrichment analysis with the hypeR R package (v.2.1.0) was used to estimate possible enrichment in biological pathways [37]. An FDR of ≤0.05 was considered as statistically significant pathway enrichment.

## 3. Results

### 3.1. Recurrent TNBC Is Characterized by Ten DE cmiRNAs

**Figure 1:**
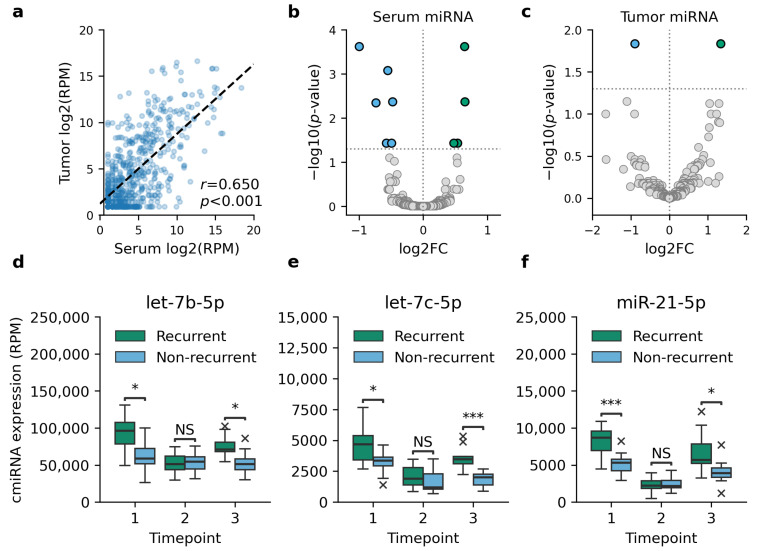
Differential expression analysis between tumor and serum samples of recurrent and non-recurrent TNBC cases. (**a**) Comparison between matched serum and tumor miRNA expression showed a moderate Pearson correlation in all cases, of which one is shown as an example. (**b**) Ten cmiRNAs were observed to be DE in recurrent TNBC at the time of diagnosis. (**c**) Two of these miRNAs were DE in matched tumor samples as well. The indicated cmiRNAs, let-7b-5p (**d**), let-7c-5p (**e**), and miR-21-5p (**f**), were DE prior to the detection of recurrent disease as well. In d–f, timepoints 1–3 refer to the time of diagnosis, follow-up sample, and the latest follow-up sample prior to the detection of recurrence, respectively. Asterisks in panels d-f indicate statistical significance levels, with * for *p* < 0.05, *** for *p* < 0.001, and NS for a non-significant result.

We detected 2656 unique miRNAs from sequenced serum and tumor samples, of which 549 miRNAs (20.7%) were expressed only in serum samples and 76 miRNAs only in tumor samples. A statistically significant correlation between tumor miRNA and cmiRNA expression levels was observed for all miRNAs with a median correlation coefficient of 0.633 (range 0.604–0.665) (Figure 1a).

Differential expression analysis identified ten cmiRNAs that were DE between recurrent and non-recurrent TNBC cases at the time of diagnosis (Figure 1b–c, Table 2). Only two of these miRNAs, let-7c-5p and miR-128-3p, were DE in matched tumor samples as well. Differential expression analysis was repeated for each timepoint to investigate how the expression levels of DE cmiRNAs reflect the clinical status of patients. While no DE cmiRNAs were identified at the first follow-up serum sample, the second follow-up sample collected at the latest follow-up examination prior to the detection of recurrence showed a differential expression of cmiRNAs let-7b-5p, let-7c-5p, and miR-21-5p (Figure 1d–f).

### 3.2. DE cmiRNAs Are Associated with RFS

Univariable survival analysis showed that the upregulated expression of cmiRNA miR-21-5p and the downregulated expression of miR-16-5p, miR-26b-5p, miR-146a-5p, and miR-199a-5p were associated with poor RFS when patients were divided into low and high-expression groups according to the median expression level (Table 3). Univariable survival analysis for BCSS and OS showed similar trends; the upregulated expression of miR-21-5p and the downregulated expression of miR-16-5p and miR-26b-5p were associated with poor BCSS and OS. In addition, the downregulated expression of miR-199a-5p and the upregulated expression of let-7b-5p were associated with poor BCSS.

Multivariable survival analysis identified miR-16-5p, miR-21-5p, and miR-26b-5p as independent prognostic factors for RFS. The upregulated expression of miR-21-5p was associated with poor RFS (*p* = 0.040, HR = 1.87, 95% CI 1.06–3.30), while the upregulated expression of miR-16-5p (*p* = 0.032, HR = 0.53, 95% CI 0.30–0.95) and miR-26b-5p (*p* = 0.023, HR = 0.52, 95% CI 0.29–0.91) were both associated with improved RFS. Multivariable survival analysis did not observe any significant association with BCSS or OS, and the observed survival trends were mainly explained by the tumor features.

### 3.3. DE cmiRNAs Are Associated with Poor Tumor Characteristics

The downregulated expression of miR-128-3p and miR-199a-5p was associated with positive lymph node status (*p* = 0.048 and *p* = 0.002, respectively, Chi-squared test). The upregulated expression of miR-30e-5p was associated with higher tumor grade (*p* = 0.040, Chi-Squared test). No further associations were identified.

### 3.4. DE cmiRNAs Are Associated with Cancer-Associated Pathways

**Figure 2:**
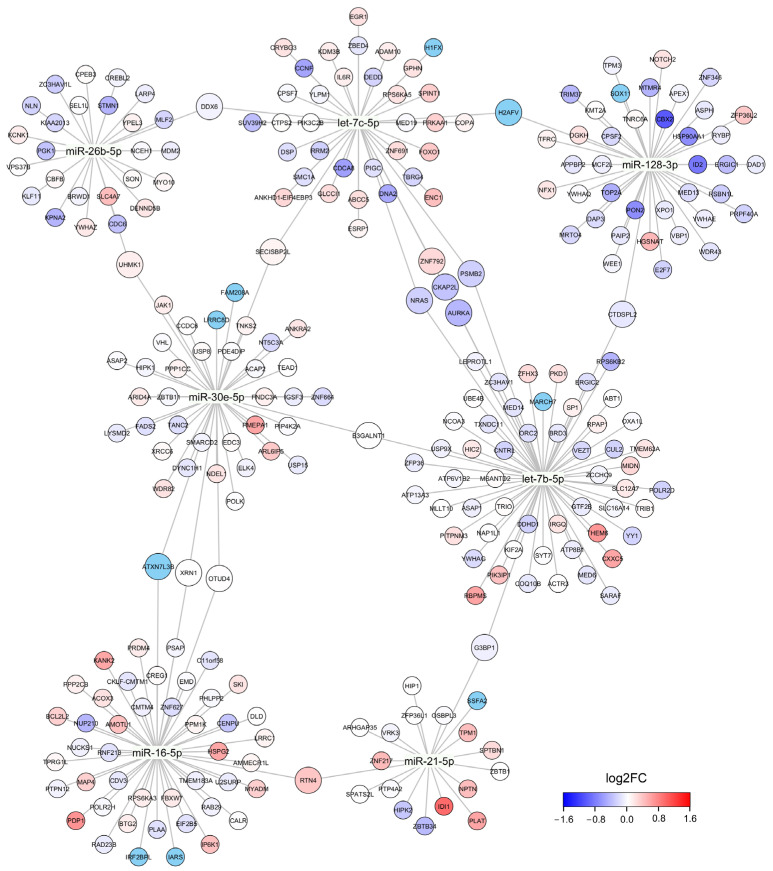
A network graph of DE cmiRNAs and their target genes. Size of the node corresponds to the number of cmiRNAs that have direct MTI with an indicated target gene. Color of the node represents fold change in differential gene expression analysis where the RNA-seq data of recurrent and non-recurrent TNBC tumor samples were compared. Only target genes that showed significant correlation with cmiRNA expression level are shown.

Our database search identified 2843 unique MTIs for DE cmiRNAs. The identified MTIs cover 1936 unique target genes, of which 89 were annotated as known tumor suppressors and 50 as known oncogenes. Correlation analysis identified 280 MTIs where the target gene expression and DE cmiRNA expression level showed significant correlation; 150 of these correlations were positive and 130 were negative (Figure 2). DE cmiRNAs miR-146a-5p, miR-199a-5p, and miR-3614-3p did not show significant correlation with their known target genes. None of these target genes were significantly DE in recurrent TNBC tumors, although some target genes showed notable fold changes.

Pathway enrichment analysis with DIANA-miRPath identified 132 significantly enriched KEGG pathways when all DE cmiRNAs were used as an input. The identified signaling pathways include many cancer-associated pathways such as pathways regulating proteoglycans, cell cycles, and p53 signaling (Table 4). Hypergeometric gene set enrichment analysis showed that the correlating target genes were significantly enriched in the cell cycle regulating pathway (*p* = 0.001), while the rest of the observed enrichments were unreliable due to there being a high FDR. These enrichments included, for example, pathways in cancer (*p* = 0.240), the TGFβ signaling pathway (*p* = 0.260), and the mTOR signaling pathway (*p* = 0.260).

### 3.5. DE cmiRNAs Improve the Performance of Logistic Regression Models

**Figure 3:**
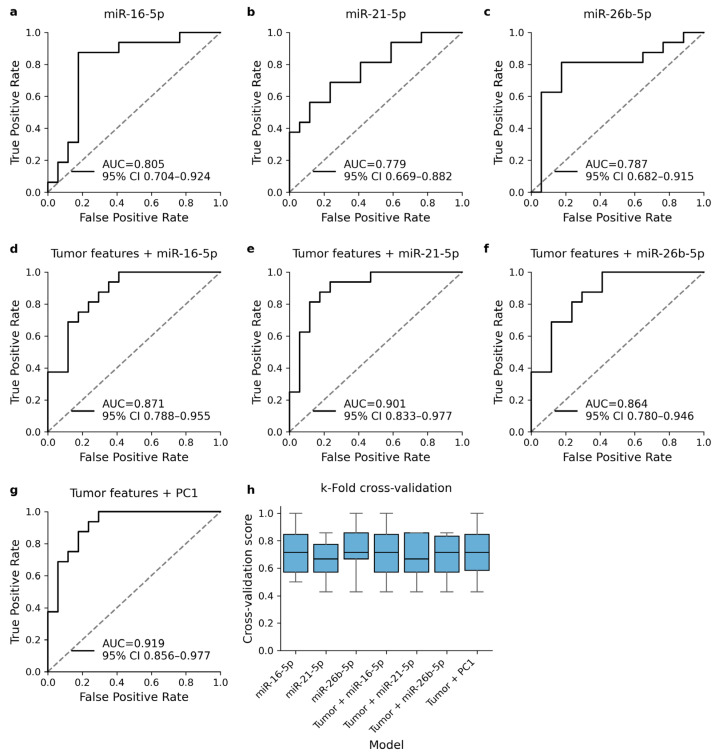
ROC analysis of logistic regression models that were trained to predict the occurrence of TNBC recurrence. Univariable regression models with DE cmiRNA expression level as the only explanatory variable (**a**–**c**) showed moderate AUC metrics. Including cmiRNA expression level into reference model improved the diagnostic performance (**d**–**f**). The best diagnostic performance was obtained when DE cmiRNA expression levels were included in the model as a component variable (**g**). All models were validated with k-fold cross-validation and showed relatively similar behavior in validation (**h**).

Univariable logistic regression models predicted the presence of recurrent disease relatively well and showed moderate AUC values in ROC analysis (Figure 3a–c). None of the univariable models were able to outperform the reference model (AUC = 0.847, 95% CI 0.762–0.955). Including the cmiRNA expression level in the analysis was found to improve the diagnostic performance of the multivariable regression models (Figure 3d–f). Including PC1 in the multivariable regression model instead of single cmiRNA expression levels was found to provide slightly better overall performance (Figure 3g). All models showed similar behavior in cross-validation (Figure 3h).

## 4. Discussion

TNBC is often considered the most aggressive subtype of BC, accounting for up to 30% of all BC deaths [38]. Although considerable achievements have been made in the molecular profiling of TNBC, only few of these prognostic and predictive biomarkers have notably improved the TNBC survival [39]. Blood-based biomarkers, such as cmiRNAs, might have the potential to identify BC patients at higher risk of recurrent disease and thus enable more proactive monitoring and treatment of these patients.

One of the key assumptions behind the concept of liquid biopsy is that tumor-specific biomarkers can be detected in body fluids. Elevated miRNA expression levels in both matched tumor and serum samples would suggest that the miRNA is tumor-specific and plays a role in tumorigenesis. Previous studies have reported significant differences and modest correlations between the expression levels of matched tumor and plasma samples [40,41,42]. Our results acknowledge similar challenges, as only a subset of detected cmiRNAs were expressed in the matched tumor samples. This suggests that the results of tumor sequencing are either affected by intra-tumoral heterogeneity and unrepresentative tumor samples or that the majority of detected cmiRNAs do not originate from the tumor tissue. This can be considered as a major challenge for the diagnostic use of cmiRNAs and emphasizes the need for careful evaluation of their biological sources. It is obvious that more extensive studies on the miRNA expression profiles of matched tumors and serum or plasma samples are required to identify factors that influence miRNA expression levels.

Our results indicate that three cmiRNAs—miR-16-5p, miR-21-5p, and miR-26b-5p, are independent prognostic factors for TNBC recurrence. Existing evidence regarding the association between these cmiRNAs and the risk of TNBC recurrence is conflicting. The most compelling evidence lies with miR-21-5p, whose overexpression is known to occur in wide range of solid tumors, including TNBC [43,44]. The upregulated expression of miR-21 has been connected to poor clinical outcome, and recent functional studies have demonstrated that miR-21 promotes tumor cell proliferation and invasion by targeting tumor suppressors *PIK3R1* and *PTEN* [45,46,47,48]. Both tumor suppressors are negative regulators of the PI3K/Akt signaling pathway, whose activation has been shown to be associated with the aggressive disease profile of TNBC [49]. The upregulation of expression of miR-21-5p has been previously connected to recurrent BC as well [50].

Experimental evidence for the functional and prognostic role of miR-16-5p and miR-26b-5p is less comprehensive. Elevated expression levels of miR-16-5p have been observed in the plasma of BC patients and predict the response to immunotherapies [51,52]. Cell studies conducted with TNBC cell lines have linked the upregulated expression of miR-16-5p to favorable cellular behavior, such as suppressed proliferation and invasiveness [53,54], thus supporting the tumor-suppressive role of miR-16-5p suggested by our results. Likewise, functional studies have reported that miR-26b-5p inhibits cell proliferation and epithelial–mesenchymal transition (EMT) in TNBC cell lines MDA-MB-468 and BT-549, thus supporting the observed tumor-suppressive role [55]. To our knowledge, the association between the upregulated serum miR-16-5p and miR-26b-5p expression and improved RFS has not been previously reported in the context of TNBC.

Performed pathway enrichment analysis with predicted MTIs suggested significant enrichment with cancer-associated biological pathways that are known to be associated with TNBC recurrence [56,57]. Since the pathway enrichment analyses from the list of miRNAs may lead to inaccurate results, we carefully validated the results by comparing the list of identified target genes to total tumor RNA-seq data and by repeating the pathway enrichment analysis with another tool [58]. Although we were unable to demonstrate differential gene expressions of the target genes in the tumor, our results indicate that target genes involved in the cell cycle regulation are potential regulatory targets of DE cmiRNAs and might, to some extent, explain the association with TNBC recurrence risk and DE cmiRNA expression.

We performed logistic regression analysis to assess the diagnostic potential of cmiRNAs in the TNBC recurrence risk assessment. Although the used dataset is undeniably too small to reliably assess the diagnostic potential of the DE cmiRNAs, our results suggest that combining cmiRNA expression data with traditional clinicopathological tumor features might improve the performance of the TNBC recurrence risk prediction model and allow the early detection of TNBC patients who might benefit from proactive monitoring.

Our study has certain limitations that should be addressed. Firstly, it is important to note that the used sample material was collected in the 1990s and has undergone long-term freezing for approximately three decades. Although cmiRNAs in general seem to withstand long-term freezing relatively well, freezing may affect the observed cmiRNA expression levels and obtained results [59,60]. To address this challenge with our sample material, the effect of long-term freezing was assessed by comparing the sequencing data to more recent sample material that was collected in 2010–2014 (Table S1). However, the potential impact of long-term freezing on sequencing results cannot be entirely excluded despite our efforts. Another time-related challenge with the sample material is the recent advancement in TNBC treatments. At the time sample collection, ITs were not available, and TNBC was often treated with standard therapies due to limited understanding of its distinct biology. Therefore, our patient material does not reflect current TNBC treatment practices, and our findings cannot be directly generalized to patients treated according to modern standards. Moreover, our study focused on Finnish TNBC patients whose genetic background is unique due to Finland’s geographic location and isolated population history. Given the heterogeneous nature of TNBC, our results may not be directly generalizable to TNBC patients with different genetic backgrounds and environmental factors.

Secondly, the shown associations with biological signaling pathways are based on database records of experimentally validated direct MTIs that have been reported in breast cancer-related studies, and we have not provided any functional validation to support our findings. Functional in vivo and in vitro experiments are needed to validate our results and elucidate the cellular mechanism behind the observed association with TNBC recurrence. The objective of our study was to identify cmiRNAs associated with TNBC recurrence, and functional experiments are a natural continuation for this study in the future.

It is evident that our results require careful validation with a separate and larger patient cohort comprising a diverse group of TNBC patients treated according to modern treatment regimens. Although our results are promising and support the diagnostic potential of cmiRNAs in predicting TNBC recurrence, they should be considered preliminary until validated in other sample sets.

## 5. Conclusions

Circulating miRNAs are considered as potential biomarkers for TNBC recurrence. Our results show that cmiRNAs miR-16-5p, miR-21-5p, and miR-26b-5p are DE in recurrent TNBC, and their expression levels are associated both with the RFS and relevant biological pathways. Our results indicate that these cmiRNAs might possess predictive value and help to identify high-risk TNBC patients who have a higher risk for developing recurrent disease after curative treatment. Future studies to validate our results are well warranted.

## Figures and Tables

**Table 1:** Patient characteristics.

Variable	Grouping	Non-Recurrent (*n* = 19)	Recurrent (*n* = 14)
Age (years)	≤39	4 (21.1)	1 (7.1)
	40–49	3 (15.8)	2 (14.3)
	50–59	6 (31.6)	5 (35.7)
	60–69	2 (10.4)	2 (14.3)
	≥70	4 (21.1)	4 (28.6)
Tumor grade	II	1 (5.3)	3 (21.4)
	III	18 (94.7)	11 (78.6)
Tumor size	T1	10 (52.6)	5 (35.7)
	T2	8 (42.1)	5 (35.7)
	T3	1 (5.3)	4 (28.6)
Lymph node status	N0	14 (73.7)	6 (42.9)
	N1	5 (26.3)	7 (50.0)
	N2	0 (0.0)	1 (7.1)
Chemotherapy	Yes	5 (26.3)	3 (21.4)
	No	14 (73.7)	11 (78.6)
Radiotherapy	Yes	9 (47.4)	4 (28.6)
	No	10 (52.6)	10 (71.4)
Hormonal therapy	Yes	3 (15.8)	6 (42.9)
	No	16 (84.2)	8 (57.1)

**Table 2:** Differentially expressed miRNAs in serum and tumor samples.

miRNA	Accession ID	Serum		Tumor	
		log_2_FC	FDR	log_2_FC	FDR
hsa-let-7b-5p	MIMAT0000063	0.54	0.037	0.43	0.661
hsa-let-7c-5p	MIMAT0000064	0.48	0.037	1.33	0.015
hsa-miR-16-5p	MIMAT0000069	−0.48	0.004	−0.01	0.993
hsa-miR-21-5p	MIMAT0000076	0.65	2.38 × 10^−4^	0.09	0.952
hsa-miR-26b-5p	MIMAT0004500	−0.55	8.39 × 10^−4^	−0.33	0.706
hsa-miR-30e-5p	MIMAT0000692	−0.50	0.037	0.25	0.844
hsa-miR-128-3p	MIMAT0000424	−0.74	0.004	−0.90	0.015
hsa-miR-146a-5p	MIMAT0000449	−0.57	0.037	0.54	0.762
hsa-miR-199a-5p	MIMAT0000231	−1.00	2.38 × 10^−4^	0.646	0.453
hsa-miR-3614-3p	MIMAT0017993	0.65	0.004	NA	NA

log_2_FC; log_2_ transformed fold change. Recurrent TNBC cases were set as a reference group in the DESeq2 analysis, and negative log_2_ fold changes represent miRNAs that were less expressed in the recurrent TNBC cases. FDR; adjusted false discovery rate

**Table 3:** Association between differentially expressed cmiRNA expression levels and RFS.

miRNA	Univariable Analysis		Multivariable Analysis		
	Z	*p*	HR	95% CI	*p*
hsa-let-7b-5p	1.61	0.204	1.23	0.70–2.16	0.301
hsa-let-7c-5p	0.62	0.423	1.16	0.65–2.04	0.616
hsa-miR-16-5p	19.96	7.91 × 10^−6^	0.53	0.30–0.95	0.032
hsa-miR-21-5p	16.82	4.12 × 10^−5^	1.87	1.06–3.30	0.030
hsa-miR-26b-5p	19.57	9.61 × 10^−6^	0.52	0.29–0.91	0.023
hsa-miR-30e-5p	0.70	0.404	0.93	0.53–1.62	0.787
hsa-miR-128-3p	3.55	0.060	0.77	0.44–1.36	0.375
hsa-miR-146a-5p	4.21	0.040	0.75	0.43–1.32	0.322
hsa-miR-199a-5p	4.67	0.031	0.76	0.43–1.34	0.341
hsa-miR-3614-3p	3.06	0.080	1.25	0.71–2.21	0.433

**Table 4:** The most enriched signaling pathways in pathway enrichment analysis. Analysis was conducted with two alternative methods; DIANA-miRPath was run with all DE cmiRNA target genes that had an existing record in Tarbase, while hypeR was run with a list of target genes that showed significant correlation with matched tumor mRNA expression levels.

KEGG Pathway Name	DIANA-miRPath ^1^	HypeR ^2^
Ubiquitin mediated proteolysis	3.97 × 10^−12^	0.130
Protein processing in endoplasmic reticulum	9.14 × 10^−12^	NA
Pathways in cancer	1.39 × 10^−11^	0.240
Shigellosis	1.39 × 10^−11^	NA
Adherens junction	6.75 × 10^−11^	1.000
Autophagy-animal	7.51 × 10^−11^	NA
Proteoglycans in cancer	1.10 × 10^−10^	NA
Cell cycle	4.46 × 10^−10^	0.001
FoxO signaling pathway	1.64 × 10^−8^	NA
Hepatitis B	2.02 × 10^−8^	NA
p53 signaling pathway	2.02 × 10^−8^	0.860
Neurotrophin signaling pathway	8.63 × 10^−8^	0.006
Hippo signaling pathway	9.16 × 10^−8^	NA
TGF-beta signaling pathway	9.16 × 10^−8^	0.260
Prostate cancer	1.00 × 10^−7^	0.260
Focal adhesion	2.00 × 10^−7^	1.000
Salmonella infection	3.85 × 10^−7^	NA
Tight junction	2.40 × 10^−6^	0.860
Rap1 signaling pathway	2.40 × 10^−6^	NA
Oocyte meiosis	2.94 × 10^−6^	0.001

^1^ Conducted for target genes of DE cmiRNAs with an existing record in Tarbase. ^2^ Conducted for target genes whose gene expression correlated with DE cmiRNA expression. NA; Significance not available.

## Data Availability

The datasets used and analyzed during the study are available from the corresponding authors on reasonable request.

## Supplementary Materials

The following supporting information can be downloaded at https://www.mdpi.com/article/10.3390/cells13221884/s1, Table S1: Differentially expressed cmiRNAs in cmiRNA samples that were isolated from older KBCP and more recent ILRS serum samples to estimate the effect of long-term freezing on cmiRNA expression levels.
