# Supplementary material for: Circulating Micro-RNAs Predict the Risk of Recurrence in Triple-Negative Breast Cancer
Source: Cells. 2024 Nov 14;13(22):1884. doi: 10.3390/cells13221884 (PMC11593277; doi:10.3390/cells13221884)
Supplement: Supplementary file 1 [file cells-13-01884-s001.zip › cells-3265110-supplementary.pdf]

Supplementary Table 1. Differentially expressed cmiRNAs in serum samples from older KBCP and more recent ILRS cohorts. Samples were compared to estimate the effect of long-term freezing on cmiRNA expression levels.

miRNA	Accession ID	log2FC	FDR
hsa-let-7i-3p	MIMAT0004585	−5.45	0.019
hsa-miR-10b-5p	MIMAT0000254	−1.22	0.011
hsa-miR-15a-5p	MIMAT0000068	−5.29	0.007
hsa-miR-19b-3p	MIMAT0000074	−2.65	8.570×10^{-5}
hsa-miR-34a-5p	MIMAT0000255	−3.88	0.012
hsa-miR-95-3p	MIMAT0000094	−6.63	0.004
hsa-miR-99b-5p	MIMAT0000689	−2.75	0.037
hsa-miR-100-5p	MIMAT0000098	−5.31	0.001
hsa-miR-130a-3p	MIMAT0000425	−4.47	0.001
hsa-miR-141-5p	MIMAT0004598	−6.95	0.001
hsa-miR-144-5p	MIMAT0004600	−5.60	0.001
hsa-miR-150-5p	MIMAT0000451	1.64	0.011
hsa-miR-155-5p	MIMAT0000646	1.46	2.791×10^{-4}
hsa-miR-182-5p	MIMAT0000259	−2.91	0.004
hsa-miR-196a-5p	MIMAT0000226	−5.93	0.011
hsa-miR-197-3p	MIMAT0000227	4.88	3.174×10^{-4}
hsa-miR-200a-3p	MIMAT0000682	−5.46	7.431×10^{-4}
hsa-miR-200a-5p	MIMAT0001620	−6.43	4.606×10^{-2}
hsa-miR-301a-3p	MIMAT0000688	−5.24	2.230×10^{-4}
hsa-miR-328-3p	MIMAT0000752	4.26	4.247×10^{-4}
hsa-miR-340-5p	MIMAT0004692	−1.73	0.014
hsa-miR-361-3p	MIMAT0004682	−2.75	0.005
hsa-miR-374a-3p	MIMAT0004688	−5.49	0.005
hsa-miR-374a-5p	MIMAT0000727	−3.70	0.013
hsa-miR-382-5p	MIMAT0000737	3.67	0.001
hsa-miR-423-3p	MIMAT0001340	2.91	0.005
hsa-miR-455-5p	MIMAT0003150	−5.94	0.004
hsa-miR-532-5p	MIMAT0002888	−2.20	0.002
hsa-miR-542-3p	MIMAT0003389	−6.24	7.567×10^{-5}
hsa-miR-574-3p	MIMAT0003239	−3.72	0.001
hsa-miR-574-5p	MIMAT0004795	−5.28	0.0290
hsa-miR-584-5p	MIMAT0003249	6.22	0.013
hsa-miR-1255b-5p	MIMAT0005945	6.88	0.006
hsa-miR-1306-5p	MIMAT0022726	4.12	0.024
hsa-miR-2110	MIMAT0010133	5.18	0.026
hsa-miR-3529-3p	MIMAT0022741	−2.75	0.005

log2FC; log2 transformed fold change. KBCP samples were set as a reference group in the DESeq2 analysis, and negative log2 fold changes represent miRNAs that were less expressed in the KBCP samples. FDR; Adjusted false discovery rate
